# Assessing an Internet-Delivered, Emotion-Focused Intervention Compared With a Healthy Lifestyle Active Control Intervention in Improving Mental Health in Cancer Survivors: Protocol for a Randomized Controlled Trial

**DOI:** 10.2196/36658

**Published:** 2022-07-27

**Authors:** Isabelle S Smith, Rebecca Wallace, Cornelia Wellecke, Marie-Abèle Bind, Karen L Weihs, Bei Bei, Joshua F Wiley

**Affiliations:** 1 School of Psychological Sciences and Turner Institute for Brain and Mental Health Monash University Melbourne Australia; 2 Biostatistics Center Massachusetts General Hospital Boston, MA United States; 3 Department of Psychiatry College of Medicine University of Arizona Tucson, AZ United States; 4 Peter MacCallum Cancer Centre Melbourne Australia

**Keywords:** cancer survivor, depressive symptoms, anxiety symptoms, emotion regulation, Unified Protocol, transdiagnostic, internet-delivered intervention, quality of life, eHealth, randomized controlled trial, psycho-oncology, mobile phone

## Abstract

**Background:**

Cancer survivors are vulnerable to experiencing symptoms of anxiety and depression and may benefit from accessible interventions focused on improving emotion regulation. CanCope Mind (CM) was developed as an internet-delivered intervention adapted from the Unified Protocol for Transdiagnostic Treatment of Emotional Disorders to improve emotion regulation and support the mental health of cancer survivors.

**Objective:**

This protocol aims to provide an outline of the CanCope Study, a trial comparing the efficacy of a Unified Protocol–adapted internet-delivered intervention (CM) designed for cancer survivors compared with an active control condition—an internet-delivered healthy lifestyle intervention, CanCope Lifestyle (CL). The primary aim is to assess and compare the efficacy of both interventions in improving emotion regulation, anxiety and depressive symptoms, and quality of life. The secondary aims involve assessing the mechanisms of the CM intervention.

**Methods:**

This trial is a 2-arm randomized controlled trial that allocates cancer survivors to either CM or CL. Both interventions comprise 4 web-based modules and are expected to take participants at least 8 weeks to complete. Participants’ mental and physical health will be assessed via self-reported surveys at baseline (T_0_), between each module (T_1_, T_2_, and T_3_), immediately after the intervention (T_4_), and at 3-month follow-up (T_5_). The study aims to recruit 110 participants who have completed T_4_.

**Results:**

The CanCope study began recruitment in September 2020. A total of 224 participants have been randomized to the CM (n*=*110, 49.1%) and CL (n=114, 50.9%) groups.

**Conclusions:**

This is one of the first trials to develop and investigate the efficacy of a web-based intervention for cancer survivors that specifically targets emotion regulation.

**Trial Registration:**

Australian Clinical Trials ACTRN12620000943943; https://tinyurl.com/b3z9cjsp

**International Registered Report Identifier (IRRID):**

DERR1-10.2196/36658

## Introduction

### Background

The end of cancer treatment is a challenging transition for many cancer survivors. Individuals can continue to experience cancer-related distress long after primary treatment has ended. Specifically, 42% and 29% of cancer survivors experience at least subclinical symptoms of anxiety and depression, respectively, and worse mental health compared with the general population [1-3]. Even 10 years after a diagnosis, large population-based studies have shown that cancer survivors are at an elevated risk of experiencing anxiety and depressive symptoms compared with cancer-free, age-matched controls [4]. Advances in diagnosis and treatment have resulted in an increasing number of individuals surviving cancer. Therefore, there is a need to support the ongoing mental health of cancer survivors.

Emotion regulation can be defined as the ability to adapt to one’s affective experience, such as maintain, increase, or decrease feelings, behaviors, or physiological responses that comprise an emotional experience [5]. Difficulty with emotion regulation, or emotion dysregulation, is a core transdiagnostic feature underlying the development and maintenance of multiple psychopathologies, including anxiety and depression [6,7]. Emotion regulation capacities may account for one’s ability to manage stressful life events, including cancer [8-10]. Strategies for regulating emotions can be categorized as either avoidance-oriented (ie, disengagement-based, such as expressive suppression, experiential avoidance, and denial) or approach-oriented (ie, engagement-based, such as cognitive reappraisals, problem solving, emotional expression, and acceptance). Avoidance-oriented strategies are associated with higher rates of emotional distress than approach-oriented strategies, including anxiety and depressive symptoms among cancer survivors [11-14]. Therefore, upregulating adaptive emotion regulation processes may be an effective way of improving mental health outcomes among cancer survivors.

One cognitive-behavioral intervention designed to target approach-oriented emotion regulation is the Unified Protocol (UP) [15]. The UP is considered transdiagnostic as it effectively improves emotional distress, including symptoms of anxiety and depression, across a range of psychopathologies [16]. Furthermore, pilot studies have demonstrated that the UP may improve depressive symptoms and emotion regulation among people with breast cancer [17]. This suggests that the UP may be effectively adapted for oncological populations. On the basis of these findings, we recently developed the CanCope intervention, an internet-delivered adaptation of the UP’s modules, tailored to the needs of cancer survivors of any diagnosis. Each CanCope module was assessed independently in a series of pilot trials. Our results suggested high feasibility, participant satisfaction, and preliminary efficacy of each independent module in improving emotion regulation and mental health symptoms when delivered remotely [18]. These are promising preliminary findings, given that accessibility to face-to-face mental health support for oncological populations is often comprised of geographic location, financial strain, lack of available health care personnel, the iatrogenic effects of cancer treatments, and more recently the COVID-19 pandemic [1,19-24]. This inequality in care indicates the need for evidence-based, remotely delivered, and scalable psychological interventions for cancer survivors.

Although many psychological interventions target the mental health of patients with cancer and cancer survivors [25-27], few have examined the potential underlying mechanisms. This is not specific to psycho-oncology; the lack of well-designed mechanistic research is widespread across intervention research [28,29]. Overall, there is a lack of understanding around *why* and *how* interventions achieve their desired effects. For instance, meta-analytic findings reveal that even though the UP may be effective in improving symptom outcomes, there is a lack of evidence to suggest that these effects are mediated through changes in emotion regulation [16].

Similarly, little is known about the effectiveness of *specific* intervention components, information critical for understanding the causal mechanisms that drive improvements in mental health [30]. Uncovering intervention mechanisms and active components is required to optimize future intervention designs and maximize treatment efficiency and outcomes [29,31]. The UP allows for the assessment of mechanisms, as the protocol adopts a modular approach, where each module targets a distinct emotion regulation skill (eg, identifying and understanding emotions, mindful acceptance of emotions, cognitive reappraisals, and experiential avoidance). One small study assessed the effects of each of these modules when delivered in-person, and the findings support the isolated therapeutic effects of each UP component [32]. The authors’ CanCope pilot studies extended these findings, showing that when delivered via the internet, each independently delivered module was associated with improvements in the intended module-specific outcomes [18]. For example, unhealthy beliefs about emotions reduced after participating in the CanCope Understanding Emotions module, and levels of mindfulness increased after taking part in the CanCope Mindful Emotion Awareness module.

In addition, evidence suggests that the UP may improve broader health outcomes and overall quality of life (QoL) [16,33-35]. Compared with the general population, cancer survivors experience inferior QoL. Indeed, up to 75% of survivors experience iatrogenic health deficits associated with reduced QoL and length of survival [22,36]. Emotion regulation is plausibly related to QoL among cancer survivors [37]. How people with cancer regulate emotions is associated with both the physiological and psychological adaptation to cancer, which can in turn impact QoL and disease prognosis [38]. Thus, the UP-adapted and cancer-specific CanCope intervention may not only improve emotion regulation but also overall QoL. By assessing intervention effects on QoL, and not simply deficits in mental health, a more holistic and meaningful perspective of health can be examined.

### Aims and Hypotheses

To address these limitations, a 2-arm randomized controlled trial was designed to assess the efficacy of an internet-delivered, multimodular UP-based intervention package with all modules combined, titled CanCope Mind (CM). CM was compared with a healthy lifestyle active control intervention, CanCope Lifestyle (CL).

### Primary Aim

Aim 1 is to assess the efficacy of CM versus CL in reducing emotion dysregulation (primary outcome) and in improving anxiety and depressive symptoms and QoL (secondary outcomes) in cancer survivors. Hypothesis 1 states that, compared with CL, after treatment (T_4_), cancer survivors randomized to CM will experience fewer difficulties regulating emotions as well as lower symptoms of anxiety and depression. Both CM and CL are expected to improve QoL, as CL includes components that target diet, physical activity, relaxation, and sleep. Given that these 4 lifestyle factors are associated with improved QoL, it is unclear whether CM or CL will be more effective in improving QoL.

### Secondary Aims

#### Aim 2 (Exploratory)

Aim 2 is to explore the role of each of the CM modules and whether the intervention is associated with changes in module-specific target outcomes (see Table 1 for CM modules and their associated outcomes).

**Table 1:** CM^a^ module outline and outcomes.

Module	Description	Module-specific outcomes
1. Understanding emotions	Part 1 (2 days): learn about the adaptive functions of emotions. Day 1^b^ is a core activity. Part 2 (2 days): learn about unhelpful beliefs about emotions. Part 3 (10 days): learn about the 3-component model of emotional experiences (thoughts, feelings or physical sensations, and behaviors). Explore each component in one’s daily life. Day 5^b^ is a core activity.	Decrease unhelpful beliefs about emotions (BES^c^ [39])
2. Mindful emotion awareness	Part 1 (7 days): learn about mindfulness and nonjudgment of emotions. Practice mindfulness using daily 10-min guided audios. Day 1^b^ is a core activity. Part 2 (7 days): practice daily “anchoring” techniques to ground oneself in the present moment. Day 8^b^ is a core activity.	Increase mindfulness skills (SMQ^d^ [40])
3. Flexible thinking	Part 1 (14 days): learn about common “thinking traps” (ie, cognitive distortions such as catastrophizing) and how to challenge cognitive distortions. Practice daily cognitive reappraisal exercises to develop balanced thinking patterns. Day 1^b^ is a core activity.	Increase use of cognitive reappraisal strategies (CERQ^e^, UP-CSQ^f^ [32,41])
4. Doing things differently	Part 1 (6 days): learn about the impact of unhelpful EDBs^g^ (eg, avoidance) in perpetuating negative thoughts or emotions. Practice identifying one’s own EDBs. Day 1^b^ is a core activity. Part 2 (8 days): learn about the importance of challenging EDBs. Practice replacing unhelpful EDBs with healthier alternative actions (eg, approach-oriented rather than avoidance-oriented behaviors). Day 7^b^ is a core activity.	Decrease experiential avoidance (MEAQ-30^h^ [42])

^a^CM: CanCope Mind.

^b^Each module comprises 1 to 2 *core activities*, which must be completed for participants to finish the module and move on to the next module.

^c^BES: Beliefs About Emotions Scale.

^d^SMQ: Southampton Mindfulness Questionnaire.

^e^CERQ: Cognitive Emotional Regulation Questionnaire.

^f^UP-CSQ: Unified Protocol–Cognitive Skills Questionnaire.

^g^EDBs: emotion-driven behaviors.

^h^MEAQ-30: Multidimensional Experiential Avoidance Questionnaire-30.

#### Aim 3

If the CM intervention improves emotion dysregulation, we will assess whether changes in emotion dysregulation mediate the effects of CM versus CL on anxiety and depressive symptoms. Hypothesis 2 states that improvements in anxiety and depressive symptoms for CM compared with CL participants —after the treatment will be mediated by greater reductions in emotion dysregulation in CM participants than in CL participants.

## Methods

### Study Design and Procedure

**Figure 1:**
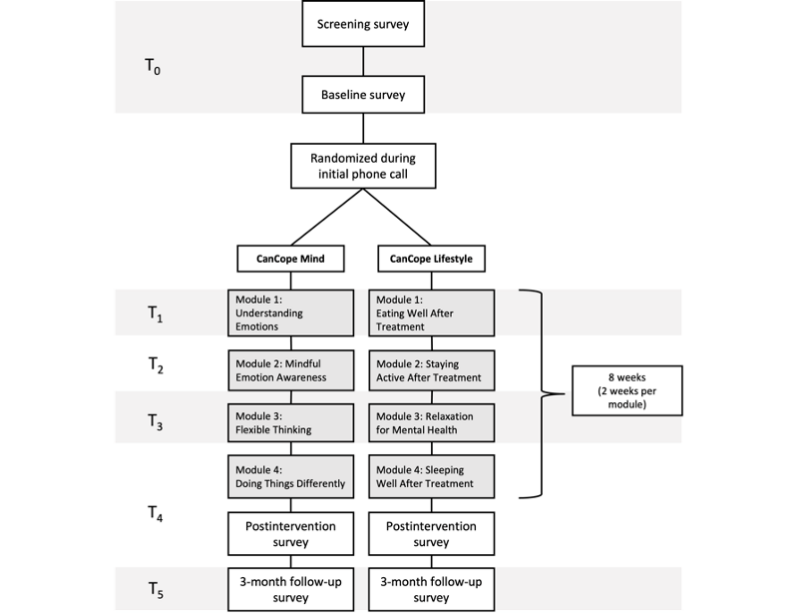
Procedure and participant flow diagram. T0: baseline; T1: post-module 1; T2: post-module 2; T3: post-module 3; T4: post-intervention and post-module 4; T5: 3-month follow-up assessment.

This trial is a 2-arm randomized controlled trial. The subsequent sections provide a detailed description of the trial procedures, and Figure 1 provides a visual summary of the assessment time points and participant flow.

#### Participants and Eligibility Criteria

Participants comprise individuals who have completed their primary cancer treatment (ie, cancer survivors; Textbox 1).

**Inclusion and exclusion criteria**

**Inclusion criteria**

- Able to read and write in English
- Able to provide informed consent
- Living in Australia, New Zealand, the United Kingdom, the United States, or Canada
- Aged ≥18 years
- Previous diagnosis of cancer (any cancer type)
- ≤2 years since finishing primary cancer treatment (ie, surgery, chemotherapy, and radiotherapy)
- Regular access to the internet and email
- Regular access to a computer, laptop, or smartphone device.

**Exclusion criteria**

- Currently undergoing or planning to undergo further primary cancer treatment (ie, surgery, chemotherapy, or radiotherapy)
- Cancer is not in remission or is progressing in severity
- Endorses current suicidality and considered high risk for self-harm
- Current episode of psychosis
- Attending regular sessions with a mental health professional (ie, attended sessions with a psychologist or counselor over the past 4 weeks or have scheduled sessions with a psychologist or counselor throughout the 8-week trial)
- Started or changed psychotropic medication or dose within the previous 2 weeks, or plans to start or change psychotropic medication or dose throughout the 8-week trial
- Previously participated in the CanCope pilot trials

No minimum criterion was included for the primary outcomes of emotion dysregulation, as it is not certain that this UP-adaption is only effective in highly dysregulated cancer survivors. By excluding participants based on a minimum cut-off, we would not be able to assess the intervention effects on cancer survivors experiencing high and low difficulties.

#### Recruitment and Consent

Participants are recruited using multiple methods, including social media platforms (eg, Facebook groups), web-based community forums (eg, Cancer Council), and via organizers of cancer support groups. Embedded within each of these advertisements, participants can access a link to the study’s written explanatory statement and consent form hosted on the Research Electronic Data Capture (REDCap) tool [43,44]. Informed consent is obtained by submission of a consent form on the web.

#### Screening and Initial Phone Call

Consenting participants are automatically redirected to a web-based screening survey to assess eligibility. Subsequently, eligible participants complete the baseline survey and are telephoned to administer the Mini International Neuropsychiatric Interview (MINI) [45] and conduct a risk assessment. Individuals deemed high risk (ie, probable risk of harming themselves) are excluded over the phone and referred to relevant psychiatric services. Eligible participants are randomized to either CM or CL. This group allocation and a summary of their intervention are conveyed to participants over the phone.

#### Randomization and Blinding

Randomization to CM or CL is conducted using a stratified, block randomization scheme generated in advance and uploaded to REDCap. Variable block sizes (4, 6, or 8) are used to ensure allocation concealment and prior guessing of the allocation sequence at the end of each block. Randomization is stratified by baseline depressive and anxiety symptoms as measured by the Patient-Reported Outcomes Measurement Information System (PROMIS) Anxiety and Depression scales (2 strata: ≥60 for either depression or anxiety, <60 for both depression and anxiety) and the Difficulties in Emotion Regulation Scale–Short Form (2 strata: <45 and ≥45). The randomization scheme is generated and set up in REDCap by a member of the research staff who is not involved in the recruitment or delivery of the intervention nor in the subsequent statistical analysis. The participants are aware of their intervention allocation (ie, CM or CL); thus, they are not blinded. However, participants remain unaware of the study hypotheses regarding which group will improve more on outcomes, and both treatments are presented as potentially effective.

### Interventions

Both the CM and CL intervention conditions are internet-delivered programs comprising 4 modules (2 weeks of content per module), which may take participants as few as 8 weeks to complete. Both interventions comprise educational readings and videos followed by activities on the web. The activities include textboxes for participants to provide written responses to encourage active reflections. All intervention materials and activities are delivered on the web via REDCap. Multimedia Appendix 1 illustrates examples of visual snapshots of activities in each intervention. All web-based activity responses completed by the participants are available for researchers to view, allowing intervention adherence to be assessed. At the beginning of each intervention and to enhance motivation, all participants are encouraged to set treatment goals. To improve engagement and provide additional support, all participants are offered an optional midintervention phone call (approximately 15 minutes) between modules 2 and 3. Upon completion of T_5_, all participants receive the alternative group’s intervention material via an email link (ie, combined psychoeducational readings and video links).

#### Treatment Group: CM

CM is a web-based version of the UP’s Transdiagnostic Treatment for Emotional Disorders [15]. The overall goal of the UP is to help individuals understand and recognize their emotions and respond to uncomfortable emotions in a more adaptive manner. Participants allocated to CM receive four UP-adapted modules titled (1) understanding emotions, (2) mindful emotion awareness, (3) flexible thinking, and (4) doing things differently. CM is a reduced format of the UP, as 2 core UP modules (modules 6 and 7) have been excluded from CM owing to their sole focus on exposure therapy, which was deemed inappropriate for remote delivery for individuals experiencing chronic health conditions. In CM, each module comprises 14 daily activities on the web (3-10 minutes each), which allow participants to apply what they have learned from the psychoeducational readings and videos. Participants are not expected to complete *every* daily activity, although they must complete at least 1 to 2 core activities per module before they could move on to the subsequent module. This format aims to mirror a more real-life, clinician-led intervention, where clients would not prematurely progress to a subsequent and more advanced module without first being exposed to fundamental skills from the previous modules (ie, in CM, participants must first learn about what an emotion is [module 1] before they can begin to mindfully engage with their emotional experiences [module 2] and change them [modules 3 and 4]). As a result, participants who reach the end of the entire intervention have been exposed to all core skills. Each module is outlined in subsequent sections, and a summary overview is provided in Table 1.

#### CM Module 1: Understanding Emotions

Module 1 aims to build an awareness and understanding of emotions through 3 psychoeducational readings, 2 summary videos, and 14 daily activities. *Part 1* explains that emotions are neither *good* nor *bad*, and it outlines the various adaptive functions of common emotions. For instance, anxiety elicits fight-or-flight responses, which is important in times of threat. During the daily activities, participants are encouraged to reflect on their own emotions and their adaptive functions in their lives. *Part 2* describes common unhelpful beliefs or misconceptions around emotions (eg, “showing emotions is a sign of weakness” and “I ‘should’ feel a certain way in particular situations”). The daily activities encourage participants to explore their own unhelpful beliefs about their emotions. *Part 3* describes the three interrelated components that constitute an emotional experience: (1) thoughts, (2) feelings or bodily sensations, and (3) behaviors. During the daily activities, participants are encouraged to bring awareness to these 3 components in their lives.

#### CM Module 2: Mindful Emotion Awareness

Module 2 aims to increase mindful awareness of emotions through 2 psychoeducational readings, 2 summary videos, and 14 activities. In *part 1,* participants are encouraged to adopt an accepting and nonjudgmental stance toward primary emotions, as critical judgment of primary emotional responses can perpetuate negative affective states. Participants are provided with 2 emotion-focused guided mindfulness audios and are encouraged to listen to one of them every day for 7 days. *Part 2* teaches a four-step mindful *anchoring* exercise: (1) paying attention to a cue, such as the breath; (2) identifying their thoughts, feelings, and behaviors; (3) considering whether their response is consistent with the present moment; and (4) shifting their response to align with the demands of the present moment. For 7 days, participants are encouraged to practice *anchoring* in *real time*, when experiencing uncomfortable emotions.

#### CM Module 3: Flexible Thinking

Module 3 aims to increase skills in *flexible thinking* (ie, cognitive flexibility). Flexible thinking involves the ability to (1) identify automatic and unhelpful thoughts and interpretations and subsequently (2) identify an alternative, more balanced appraisal of the given situation. The module comprises 1 reading, 1 summary video, and 14 activities that outline the bidirectional relationship between thoughts and emotions. Participants are taught about common and unhelpful *thinking traps* (ie, cognitive distortions), including catastrophizing, jumping to conclusions, tunnel vision, “should” statements, and all-or-nothing thinking. The daily activities on the web encourage participants to (1) identify their thinking traps, (2) answer multiple questions to challenge the thinking trap (eg, “What evidence supports my negative belief?” “Am I 100% sure these negative outcomes will occur?”), and (3) generate an alternative and more balanced appraisal of the situation.

#### CM Module 4: Doing Things Differently

The final module aims to build skills in identifying and altering unhelpful emotion-driven behaviors (EDBs) through 2 psychoeducational readings, 2 summary videos, and 14 activities. Unhelpful EDBs are defined as maladaptive ways to try to manage one’s emotions, often with the purpose of eliminating an emotion or preventing oneself from feeling an emotion in the first place. *Part 1* focuses on overt and covert avoidance-oriented EDBs (eg, avoiding certain situations or people, procrastinating, denial, ruminating, suppression, and safety behaviors) and their paradoxical effect in increasing negative emotions, such as anxiety. Two other broad categories of EDBs are discussed: reassurance-seeking behaviors (eg, excessive bodychecking for signs of cancer recurrence or excessively seeking external validation and compliments) and defensive behaviors (eg, directing anger and frustration toward others). In activities on the web, participants are encouraged to identify and reflect on these EDBs throughout their lives. *Part 2* teaches participants about *alternative actions*, value-consistent and healthier behaviors that can replace unhelpful EDBs (eg, engaging in conversations with loved ones regarding their cancer journey as opposed to avoiding cancer-related discussions owing to anxiety). In daily activities on the web, participants are encouraged to practice replacing their EDBs with alternative actions.

**Rationale for Choice of Control Group**

To assess the effects of CM on an appropriate comparator, we consulted the National Institute of Health’s Pragmatic Model for Comparator Selection in Health-Related Behavioral Trials [46]. According to the model, the best comparator is one that suits the goals of the research trial. Given that (1) the CanCope modules have demonstrated promising results when trialed independently in pilot studies and (2) this is the first efficacy trial to assess the effectiveness of the CM program in its entirety, this trial remains within the preliminary phases of the research process. We defined the goal of this trial as establishing how well CM compares to information currently readily available to cancer survivors seeking support to improve their general well-being.

Currently, patients with cancer and cancer survivors have access to a host of resources that aim to promote general QoL, often with a focus on diet, physical exercise, relaxation, and sleep. These resources are often disseminated freely on the web by cancer-specific (eg, Cancer Council, National Breast Cancer Foundation, Prostate Cancer Foundation Australia) and noncancer-specific (eg, Mind UK, Beyond Blue, and Black Dog Institute) community and government organizations. Therefore, two possible comparator options were considered: (1) a wait-list control condition versus (2) a basic lifestyle and well-being intervention targeting diet, exercise, sleep, and relaxation using free and highly accessible web-based resources.

Both options were evaluated against 7 key characteristics identified in the National Institute of Health model [46], which are summarized in Table 2. On the basis of these considerations, a 4-module web-based well-being or lifestyle comparator was chosen (ie, CL). CL is expected to be widely accepted by participants in comparison to a wait-list control group, which may result in high attrition rates, especially given the number of between-module surveys. Furthermore, a basic well-being program is deemed relevant and highly feasible, as we can draw on information available on the web and preexisting sleep resources used in sleep trials by researchers at Monash University. Most importantly, an active comparator controls for nonspecific components of the intervention, such as contact with researchers and expectancy or placebo effects, allowing for greater stringency in measuring the outcomes of interest. The primary limitation of using CL as a comparator is that improvements in diet, physical exercise, relaxation, and sleep may lead to sizeable improvements in secondary outcomes (ie, anxiety and depressive symptoms and QoL). However, the primary outcome of interest is emotion regulation. Given that the CL intervention does not focus specifically on emotions, we expect that any significantly meaningful improvements in emotion regulation will be smaller than those observed in the CM group.

**Table 2:** Comparison of potential comparator conditions.

Characteristic	Wait-list control (no intervention for 8 weeks).	CL^a^ (four modules: diet, exercise, relaxation, and sleep).
Acceptability	Moderate—participants will eventually be given the CM^b^ program, however, they may not be content with completing multiple study assessments during the 8-week waiting period.	High—participants will be provided with a program that targets health areas of interest. All participants will eventually receive the CM intervention material.
Feasibility	High—no intervention needs to be developed.	High—it is easy to access resources on the web to disseminate (from sites such as Cancer Council). Our research group has existing sleep hygiene information specifically designed for oncological populations.
Formidability	Low—no intervention means that outcomes should not change because of the comparator.	Moderate—improving diet, physical activity, relaxation, and sleep can have an impact on lowering depressive and anxiety symptoms and potentially emotion regulation.
Relevance	High—most cancer survivors do not receive a mental health intervention after finishing primary treatment.	High—basic well-being information regarding diet, physical exercise, relaxation, and sleep is commonly disseminated by hospitals and organizations, and is freely available on the web.
Stringency	Low—the absence of a comparator intervention would not control for other factors such as expectancy or placebo effects or contact with researchers.	High—controls for “nonspecific” intervention effects, such as expectancy and placebo effects and contact with researchers. The 4 comparator modules would align with the 4 CM modules, thus closely matching the treatment intervention’s timing of modules.
Uniformity	Low—participants would not receive the same information as the treatment group during the assessment period.	High—participants in the comparator group would receive a parallel 4-module program and concurrent assessment surveys.

^a^CL: CanCope Lifestyle.

^b^CM: CanCope Mind.

#### Control Group: CL

In parallel to the CM modules, the CL group receives four internet-delivered modules that focus on different lifestyle domains: (1) diet, (2) physical activity, (3) relaxation, and (4) sleep. Throughout each 2-week module, participants are sent 2 activity links via email, whereby they are asked to apply what they have learned from the module (eg, *describe how you applied the module material to your life* *over the past week* and *describe how you plan to apply the module material to your life over the next week*). Participants may take 3 to 10 minutes to complete each reflective activity on the web. Although the CL participants are sent fewer application activities on the web than the CM participants, they are still encouraged to apply the module material to their daily lives over the 2-week module duration. The content included in CL is publicly available, except for sleep hygiene information, which was developed by the Monash University sleep research group. The content of each module is outlined in the subsequent sections.

#### CL Module 1: Eating Well After Treatment

Module 1 comprises 1 video and 3 readings. The video was developed by the organization *Mind* (a mental health charity in the United Kingdom established by the National Association for Mental Health) and outlines 8 tips to improve well-being through healthy diet habits (eg, eat regularly, eat healthy fats, keep hydrated, and eat a variety of healthy vegetables). All the 3 readings were developed by the Cancer Council. The readings promote eating a healthy diet rich in fruits, vegetables, and whole grains to maintain well-being and reduce cancer risk and provide healthy recipes. Participants are encouraged to apply the healthy eating habits across the 2-week module.

#### CL Module 2: Staying Active After Treatment

Module 2 comprises 1 video and 1 reading. The video was developed by the organization *Mind* and outlines tips to encourage physical activity (eg, starting small and adhering to a consistent routine). Participants are directed to the Cancer Council’s reading; “Exercise for people living with cancer,” which provides various exercises related to strength training, aerobic exercise, flexibility, and strengthening the pelvic floor. Participants are encouraged to choose 2 exercises listed in the reading (eg, strength training) to apply throughout the module.

#### CL Module 3: Relaxation for Mental Health

Module 3 comprises educational material (1 video and 1 reading) and a link to various guided relaxation audios. The 5-minute YouTube video was developed by the organization *Mind* and outlines 8 tips to aid relaxation (eg, scheduling regular breaks, diaphragmatic breathing, visualization techniques, and listening to music). The reading, titled “Learning to relax,” was developed by the Cancer Council and discusses healthy ways to manage emotional stress and aid relaxation (eg, exercising, massage, and yoga). Finally, participants are sent a link to access Beyond Blue’s guided relaxation audio clips. Participants could choose from (1) breathing exercises, (2) muscle relaxation, and (3) guided visualizations. Participants are encouraged to listen to any of these audio clips as many times as they would like across the 2-week module.

#### CL Module 4: Sleeping Well After Treatment

The final module comprises 2 readings focused on sleep and fatigue. The first reading was developed by the Cancer Council and explains fatigue in the context of cancer and ways to manage fatigue. The second reading provides education on sleep (eg, what is sleep, stages of sleep, and the importance of sleep) and various sleep hygiene tips (eg, reducing caffeine intake, reducing light exposure at night, and increasing light exposure in the morning). This informational sheet was developed by researchers at Monash University. Participants are encouraged to apply the sleep hygiene tips across the 2-week module.

### Assessment Time Points and Measures

Table 3 summarizes the nature and timing of the assessments in this trial. The research outcomes are assessed via web-based surveys administered via REDCap concurrently to both the CL and CM groups at the following time points: baseline (T_0_, approximately 25 minutes), after module 1 (T_1_, approximately 10 minutes), after module 2 (T_2_, approximately 10 minutes), after module 3 (T_3_, approximately 10 minutes), after module 4 (T_4_, after the intervention, approximately 25 minutes), and at the 3-month follow-up (T_5_, approximately 25 minutes). Participants in the CL are automatically emailed a link to complete each postmodule survey approximately 14 days after starting a given module. For CM participants, the postmodule surveys are automatically scheduled to be sent only once the core activities are completed (core activities are outlined in Table 1). Participants in both CL and CM are unable to begin the subsequent module until they have completed the previous postmodule survey. If participants do not complete their postmodule surveys within 2 weeks of receiving their original survey link, they are withdrawn from the study. Any completed survey responses beyond the 2-week cut-off time point are considered invalid and excluded from the analyses.

**Table 3:** Schedule of survey assessments.

Time point				Items per time		Baseline (T_0_)^a^		Postallocation^a^								Follow-up (T_5_)^a^		
								T_1_ after module 1		T_2_ after module 2		T_3_ after module 3		T_4_ after module 4				
**Intervention groups**																		
		CM^b^	N/A^c^		N/A		✓^d^		✓		✓		✓		N/A			
		CL^e^	N/A		N/A		✓		✓		✓		✓		N/A			
**Primary outcome**																		
		DERS-SF^f^	18		✓		✓		✓		✓		✓		✓			
**Secondary outcomes**																		
		Depression (PROMIS^g^)	4		✓		✓		✓		✓		✓		✓			
		Anxiety (PROMIS)	4		✓		✓		✓		✓		✓		✓			
		QoL^h^ (PROMIS)	30		✓		N/A		N/A		N/A		✓		✓			
**Module-specific target outcomes**																		
		BES^i^	12		✓		✓		✓		✓		✓		✓			
		SMQ^j^	16		✓		✓		✓		✓		✓		✓			
		CERQ^k^	8		✓		✓		✓		✓		✓		✓			
		UP-CSQ^l^	7		✓		✓		✓		✓		✓		✓			
		MEAQ-30^m^	30		✓		✓		✓		✓		✓		✓			
**Other measures**																		
		Depression Risk Questionnaire-7	7		✓		N/A		N/A		N/A		N/A		N/A			
		Positive Affect Subscale	10		✓		N/A		N/A		N/A		✓		✓			
		Demographic and cancer information	39		✓		N/A		N/A		N/A		N/A		N/A			
		Health service use (eg, current medications, mental health treatment)	10		✓		N/A		N/A		N/A		✓		✓			
		Program evaluation (Client Satisfaction Questionnaire and open feedback)	10		N/A		N/A		N/A		N/A		✓		N/A			
		Credibility Expectancy Questionnaire	6		✓		N/A		N/A		N/A		N/A		N/A			
		Adverse events (assessed throughout)	N/A		N/A		✓		✓		✓		✓		✓			
		COVID-19 pandemic impact and distress	2		✓		N/A		N/A		N/A		✓		N/A			
		MINI^n^	5 mins		✓		N/A		N/A		N/A		N/A		N/A			

^a^Completion times: T0, T4, and T5 were 25 minutes each; T1-T3 were 10 minutes each.

^b^CM: CanCope Mind.

^c^N/A: not applicable.

^d^Measure administered at that time point.

^e^CL: CanCope Lifestyle.

^f^DERS-SF: Difficulties With Emotion Regulation Scale–Short Form.

^g^PROMIS: Patient-Reported Outcomes Measurement Information System. The PROMIS scales are all computer-adaptive tests, which means that they vary in the number of items administered depending on the participants’ prior responses.

^h^QoL: quality of life.

^i^BES: Beliefs About Emotions Scale.

^j^SMQ: Southampton Mindfulness Questionnaire.

^k^CERQ: Cognitive Emotional Regulation Questionnaire. “Catastrophizing” and “Refocus on Planning” subscales.

^l^UP-CSQ: Unified Protocol–Cognitive Skills Questionnaire.

^m^MEAQ-30: Multidimensional Experiential Avoidance Questionnaire-30.

^n^MINI: Mini International Neuropsychiatric Interview.

#### Screening

The MINI [45] is conducted in an initial phone call and used as a diagnostic tool to assess the presence of a major depressive episode (module A) and generalized anxiety disorder (module N), ruling out organic causes (module O). MINI interviews are recorded, allowing for the assessment of reliability and team discussion of complex cases. Any participant who endorses the criteria for a psychiatric illness is provided with relevant mental health and emergency support numbers.

#### Primary Outcome

The Difficulties in Emotion Regulation Scale–Short Form (DERS-SF) [47] will be used to assess six domains of emotional regulation (ie, acceptance of emotional responses, emotional awareness, emotional clarity, engagement in goal-directed behaviors, impulse control, and access to emotion regulation strategies). The DERS-SF is an 18-item self-report questionnaire that asks participants to indicate the frequency of emotion-focused behaviors and thoughts on a Likert-type rating scale, with possible responses ranging from 1 (“Almost never”) to 5 (“Almost always”). Example items include “I have difficulty making sense of my feelings” and “When I’m upset, I become out of control.” Scores can range from 18 to 90, with higher scores indicating greater emotional dysregulation. The DERS-SF indicates good concurrent validity for depression, anxiety, and self-harm measures and demonstrates good internal consistency reliability for both the overall scale (Cronbach α=.90) and each individual subscale (Cronbach α ranging from .78 to .89) [48,49]. Moreover, the full version of the Difficulties in Emotion Regulation Scale has been used in prior research to assess emotion dysregulation in oncology populations [50].

#### Secondary Outcomes

The PROMIS–computer [51,52] adaptive tests for the Anxiety and Depression scales will be used to measure symptom changes. The computer-adaptive test algorithm requires a minimum of 4 items and a maximum of 12, and the test stops when the SE is less than 0.30 [51]. The scales present the statement “over the past 7 days...” followed by items, such as “I felt uneasy” (PROMIS Anxiety) and “I felt helpless” (PROMIS Depression). Each item response is rated on a 5-point Likert-type frequency scale, ranging from 1 (“Never”) to 5 (“Always”). Raw scores for each item are summed and converted into a *T* score (population mean 50, SD 10). Higher scores indicate greater depressive or anxiety symptoms (*T*<65 = normal-to-mild; *T*≥65 = moderate to severe symptoms [53]).

The PROMIS QoL Health Utility Score [54] will be calculated using preference-based weights in PROMIS−29+2 Profile (version 2.1) [55] based on computer-adaptive testing to assess QoL. The QoL outcome score is a composite of the following seven domains captured by PROMIS: physical function, pain interference, cognitive function, depression, fatigue, sleep disturbance, and the ability to participate in social roles and activities. The QoL score ranges from −0.022 (“Dead”) to 1.0 (“Full health”) and has achieved good construct validity when measured against the Health Utility Index and the EQ-5D [56].

#### Module-Specific Target Outcomes

All the module-specific target outcomes and their respective modules are listed in Table 1. The Beliefs About Emotions Scale [39] will be used to assess negative beliefs about emotions and the impact of CM’s module 1. The Southampton Mindfulness Questionnaire [40] will be used to assess mindfulness and the impact of CM’s module 2. The Unified Protocol–Cognitive Skills Questionnaire [32] will be used to assess cognitive reappraisal skills and the impact of CM’s module 3. In addition, the Cognitive Emotional Regulation Questionnaire [41] “Catastrophizing” and “Refocus on Planning” subscales will also be used to assess the impact of CM’s module 3. The Multidimensional Experiential Avoidance Questionnaire-30 [42] will be used to assess levels of emotional (experiential) avoidance and to assess the impact of CM’s module 4.

#### Intervention Evaluation Outcomes

The Credibility Expectancy Questionnaire [57] will be administered to assess the perceived credibility and expectancy of CM versus CL before commencing the intervention. The Client Satisfaction Questionnaire [58] will be used to assess the participants’ level of satisfaction with CM and CL. Questions will assess factors such as whether the intervention has met participants’ needs and whether participants would return to the service. Higher scores indicate greater satisfaction. With regard to evaluation of treatment fidelity and reliability, because both interventions are delivered in a standardized way via the internet, no additional measures of treatment fidelity or reliability are collected. With regard to adherence, for CM, adherence is measured objectively via the number of completed application activities on the web per module. For CL, adherence is measured via self-reports of whether participants indicate that they have applied the intervention content to their lives that week in the weekly web-based activities.

#### Other Measures

The Depression Risk Questionnaire-7 [59] is a brief self-reported questionnaire developed as a clinical screening tool for patients with breast cancer at risk of depression. The Positive Affect Subscale from the Positive and Negative Affect Scales [60] will be used to assess positive emotional states (eg, attentiveness, enthusiasm, pride, and interest). Two items assessing (1) impact and (2) distress caused by COVID-19 will be administered, with participants’ self-reported responses indicated on a sliding scale from 0 to 100 (0=no distress or no impact, 100=a lot of distress or a lot of impact).

### Participant Compensation

All eligible participants who complete the baseline assessment as well as at least 2 modules and their respective postintervention assessments (ie, half of an intervention, T_1_ and T_2_) are provided with an e-gift card worth equivalent to Aus $40 (US $30) in their local currency as a token of appreciation. This compensation is provided at the end of their participation.

### Statistical Analysis Plan

#### Power Analysis

The primary end point for the trial is the immediate postintervention assessment (T_4_). As all primary and secondary outcomes are continuous, the primary analyses will be linear regressions with group as a predictor and outcome scores at baseline included as a covariate. We set the type 1 error at Cronbach α=.05 (2-tailed). A Monte Carlo simulation study was conducted to determine the power analysis and required sample size. In the simulation, we varied (1) the correlation between baseline and postintervention outcome scores (*r*) across four values (0.3, 0.4, 0.5, and 0.6) selected from the literature and our pilot research [18] and (2) the standardized mean difference (SMD) between the CM and CL groups at post intervention on outcome scores (primary end point) across 2 values (SMDs of 0.5 and 0.6). The SMDs were selected based on our pilot work [18] where we observed SMDs from before to after the intervention of approximately 0.5 to 1.0 for our primary and secondary outcomes. Our pilot did not include a control group (likely inflating the SMDs) but only tested individual modules separately (likely reducing the SMDs); therefore, we believe that a moderate SMD was reasonable, on average. The code for this Monte Carlo study is publicly available [61]. Results from 10,000 Monte Carlo simulations for each of the conditions and varying sample sizes showed that participants (n=100) with complete data at T_4_ will provide more than 80% power to detect a medium (SMD=0.5) group difference—after the intervention, even with only a moderate correlation (*r*=0.3) between baseline and postintervention outcome scores with Cronbach α=.05.

#### Data Cleaning and Sample Characteristics

Analyses will be conducted in R on an intention-to-treat basis with statistical significance set at Cronbach α=.05 for type 1 error. The tests for primary and secondary outcomes will be 2-sided. The data will be assessed for outliers. If outliers are present, we will use quantile regression to calculate a median estimate or evaluate removing or winsorizing outliers.

#### Primary Aim

Missing data are ubiquitous in research, and dropout and incomplete data (eg, dropout) are particularly common in web-only trials [62]. For aim 1 analyses, we will address missing data using multiple imputation with a fully conditional specification [63] and predictive mean matching [64]. A total of 20 imputed data sets will be generated. The primary outcome of the trial is the Difficulties in Emotion Regulation Scale (DERS-SF). The primary end point is immediately after the intervention, immediately following module 4 (T_4_). Anxiety and depression symptoms as well as overall QoL are secondary outcomes. All outcomes are continuous measures.

The primary analyses will consist of linear regressions on multiple imputed data. Each outcome variable at the primary end point (T_4_) will be included as the outcome variable in a linear regression, with group as the main predictor and the outcome variable at baseline (T_0_) and stratification factors included as covariates. Adjusted means in each group, as well as the adjusted mean difference, will be calculated. In the event of outliers, quantile regression will be used in place of linear regression and adjusted median differences at the primary end point (T_4_), and as exploratory analyses, the other time points will be estimated. Following recent research suggestions [65], we chose to provide randomization-based inferences; that is, statistical significance will be based on Fisher exact *P* values [66] from 100,000 permutations for the adjusted mean differences and uncertainty intervals will be based on 95% Fisher intervals. The sharp null hypothesis tested will be that being randomized to CM has an identical effect on participants’ outcomes as being randomized to CL.

Given the conceptual overlap between stratification factors and the outcome measured at baseline, only the stratification factors for anxiety and depression will be included in the linear regression assessment of the primary outcome (DERS-SF). For the linear regression assessment of depression and anxiety symptoms, only the DERS-SF stratification factor will be included. For the linear regression assessing QoL, both stratification factors will be included.

In addition to testing for group differences at the primary end point (T_4_), we will explore group differences at T_1_, T_2_, T_3_, and T_5_ on the primary and secondary outcomes. The overall sample mean at T_0_ will be presented for comparison.

The adjusted SMDs will be calculated as the adjusted group mean difference (CM–CL) divided by the residual SD estimated from the model. In the case of outliers, standardized median difference will be calculated. By using the residual adjusted for baseline outcome scores, this is effectively an effect size on the difference scores or for repeated measures [67].

#### Secondary Aims

The same analyses, significance tests, sharp null hypothesis, and result reporting described for the primary aim will be used to assess aim 2, the effect of CM versus CL on module-specific target outcomes. The model-specific target outcomes are presented in Table 1. Using regressions (linear if no outliers and quantile if outliers), adjusted differences between CM and CL in module-specific outcome scores will be assessed after each module and at the intervention end point (T_4_) adjusted for the score before completing the module.

If intervention effects are observed on the DERS-SF, aim 3 will involve conducting mediation analyses to assess whether greater improvements on the DERS-SF (primary outcome), in the CM versus CL condition, mediate the effects of CM versus CL on anxiety and depression symptoms (secondary outcomes).

#### Sensitivity Analyses

Planned sensitivity analyses will include (1) calculating parametric and asymptotic *P* values and CIs, (2) generalized additive models to examine whether results differ when allowing nonlinear associations between baseline and postintervention symptoms, (3) per-protocol analyses based only on participants who completed all core intervention components, and (4) assessing group differences in intervention duration and the impact of intervention completion time on outcomes.

### Ethics Approval and Adverse Events

This study received ethics approval from the Human Research Ethics Committee at Monash University (ethics ID number: 25825). Any protocol modifications will be communicated to the Human Research Ethics Committee and participants. The primary investigator will make safety and progress reports to the ethics committee at least annually and within 3 months of study completion. Adverse events will be tracked in the following ways: (1) a >10-point worsening (at any time point compared with the previous time points) in PROMIS T scores on the anxiety or depressive symptom outcomes; (2) two questions administered at both T_2_ and T_4_ to assess the side effects due to the intervention and negative experiences, such as inappropriateness or unhelpfulness of the intervention; and (3) monitoring for any unsolicited reports of adverse events or serious adverse events (eg, mental health–related hospital admissions). In the consent form, participants are informed that the survey questions, including PROMIS anxiety and depressive symptom surveys, ask sensitive information and may be confronting, triggering temporary elevations in distress for some individuals. If this occurs, all participants are provided with relevant mental health support numbers to contact, and they could report this to the researchers.

## Results

Recruitment for this trial began in September 2020 and all follow-up data were collected in April 2022. A total of 224 patients were randomized to the CM (n*=*110, 49.1%) and CL (n=114, 50.9%) groups. In total, 61 CM participants and 75 CL participants have completed the intervention and postintervention assessment surveys.

## Discussion

This manuscript outlines the protocol for a novel randomized controlled trial evaluating the efficacy of a multicomponent, emotion-focused, internet-delivered intervention (CM) compared with an active comparator intervention (CL) in improving emotion regulation, symptoms of anxiety and depression, and QoL in cancer survivors. The findings from this trial will extend previous pilot results, which suggested that each CM module may be independently effective in improving cancer survivors’ mental health and emotion regulation [18].

### Limitations

The results of this trial will be interpreted in the context of its limitations. For instance, as the treatment group’s effects will not be compared against the absence of any intervention (eg, wait-list control), we will not be able to assess whether improvements in either group are due to the actual interventions or a result of naturalistic tendencies (eg, natural improvement in mental health symptoms over time). In addition, the intervention intensity and thus the potential duration of CM and CL differ. Unlike in CL, CM includes mandatory core activities in each module, which must be completed before participants can progress to the subsequent modules. This means that CM participants are likely to take longer to complete the intervention, which could impact the outcomes and attrition rates. Finally, CM and CL are English language–based interventions, and the trial excludes those who cannot read or write in English, which may limit the generalizability of findings to English-speaking populations only.

### Strengths and Significance

Despite these limitations, this is the first randomized controlled trial to evaluate an emotion-focused, UP-adapted, and internet-delivered intervention designed for cancer survivors to improve their mental health and QoL. Considering (1) that most of those diagnosed with cancer survive their diagnosis [68], (2) that cancer survivors experience elevated symptoms of psychological distress and lower QoL [1,36], and (3) that cancer survivors may face economic and geographic barriers to accessing mental health support [21,23,69], the development of web-based and affordable interventions that are highly accessible is paramount. Finally, as CM is internet-guided, it is also expected to have a low therapist burden and is considered highly scalable.

Another strength of this study relates to its unique study design. The trial will allow not only an assessment of symptom improvement but also an evaluation of factors that account for symptom changes. Specifically, by concurrently measuring emotion regulation and psychopathological symptoms at all time points, the design will enable the assessment of more nuanced relationship patterns between these closely linked variables. Thus far, few studies assessing applications of the UP have been able to clearly decipher whether improvements in emotion dysregulation are truly mediating symptom improvements.

Furthermore, the study will allow a detailed assessment and comparison of each module’s effects in the treatment versus control conditions. This mechanistic analysis allows for greater treatment optimization. For example, if the target outcome of one module is found to contribute minimally compared with the other modules, then theoretically, the less effective module may be removed or decreased in intensity to reduce intervention duration and improve treatment efficiency.

In addition, the CL intervention material may prove to be a rather formidable control comparator. This is because lifestyle factors, such as diet [70], exercise [71], relaxation [72], and sleep [73], are highly correlated with mental health outcomes. Therefore, if CM is more efficacious than CL in improving emotion regulation and anxiety and depressive symptoms, such findings will speak to the strength and magnitude of the effects of CM.

## Appendix

Multimedia Appendix 1 Supplementary materials including visual snapshots of intervention activities.

## Abbreviations

CL: CanCope Lifestyle
CM: CanCope Mind
DERS-SF: Difficulties With Emotion Regulation Scale–Short Form
EDB: emotion-driven behavior
MINI: Mini International Neuropsychiatric Interview
PROMIS: Patient-Reported Outcomes Measurement Information System
QoL: quality of life
REDCap: Research Electronic Data Capture
SMD: standardized mean difference
UP: Unified Protocol

## Data Availability

Deidentified data for this trial will be made available once collected and analyzed within 7 years of the final publication and shared on Monash University’s web-base [74]. Data sharing is not applicable to this protocol paper, as no data sets were generated or analyzed.
