# Supplementary material for: Assessing an Internet-Delivered, Emotion-Focused Intervention Compared With a Healthy Lifestyle Active Control Intervention in Improving Mental Health in Cancer Survivors: Protocol for a Randomized Controlled Trial
Source: JMIR Res Protoc. 2022 Jul 27;11(7):e36658. doi: 10.2196/36658 (PMC9377468; doi:10.2196/36658)
Supplement: Multimedia Appendix 1 [file resprot_v11i7e36658_app1.pdf]

Supplementary Material

CanCope Mind – visual snapshot of example activities

CM Module 1.

Resize font:

Survey Queue

Part 1 - Functions of Emotions (Day 2)

Welcome to today's activity. Today you will be encouraged to reflect on the emotions that you experienced today, and their functions (same as Day 1).

Tomorrow you will be sent an Email to begin Part 2.

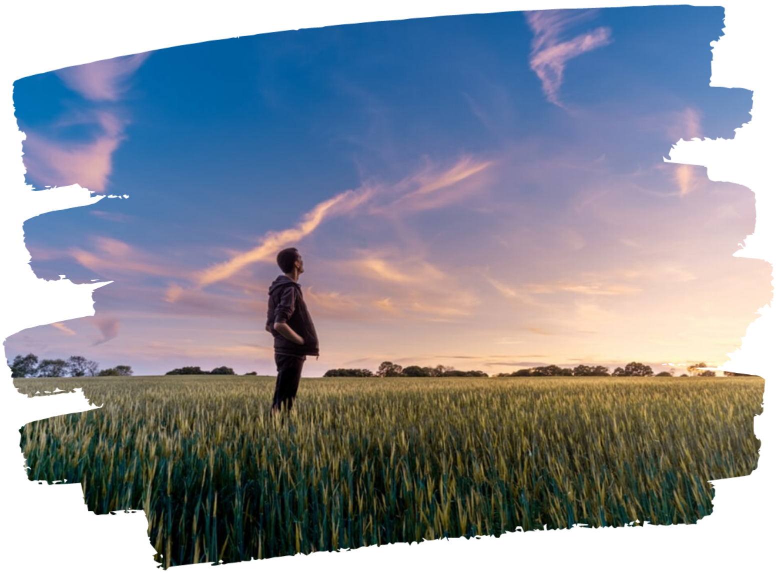

Click "**Watch video**" below to watch the summary video again.

Watch video

Click the link below if you would like to download the reading for Part 1 as a PDF.

Attachment: Part 1 - Functions of Emotions Reading.pdf (8.04 MB)

1) **What emotions did you experience today?**
(These may be uncomfortable emotions such as anxiety, sadness, or anger, or even positive emotions). If you are finding it hard to identify your emotions, refer to the emotion wheel at the bottom of this page.)
* must provide value

Expand

2) **What are the functions of these emotions in your life? i.e., How are they useful?**
(It may be helpful to consider what life would be like if you did not experience these emotions at all. We tend to think that our life would be better if we could rid ourselves of certain emotions, however, this is not really the case.)
* must provide value

CM Module 2.

Part 2 - Anchoring (Day 9)

Resize font:

Survey Queue

Spend a few minutes reflecting on your experience anchoring yourself today. You might prefer to reflect at the end of the day after anchoring yourself at least once. Keep in mind that anchoring can be done *anywhere at any time*. Some days, anchoring may take you 20 seconds, other days it may take you 4 minutes and multiple attempts!

Here is a quick reminder of the 4 steps to anchoring:

1. **Focus on a cue in your current environment (e.g., breath) to allow yourself to pause...**
2. **Do a '3-point check' ("*what am I thinking, feeling, and doing?*")...**
3. **Ask yourself whether your current experience is consistent with the present moment? (i.e. *"Am I present in the current moment or am I being swept up by past events or future worries?"*)...**
4. **If there is an inconsistency, try to change your response so that you are present in the moment. Examples of engaging in the present moment include noticing objects or sounds in your environment, engaging fully with the company of others, or focusing fully on one task.**

Click on the PDF link below if you would like to review the reading material again:

Attachment: Part 2 - Anchoring Reading.pdf (2.77 MB)

Click "Watch video" if you would like to re-watch the summary video:

Watch video

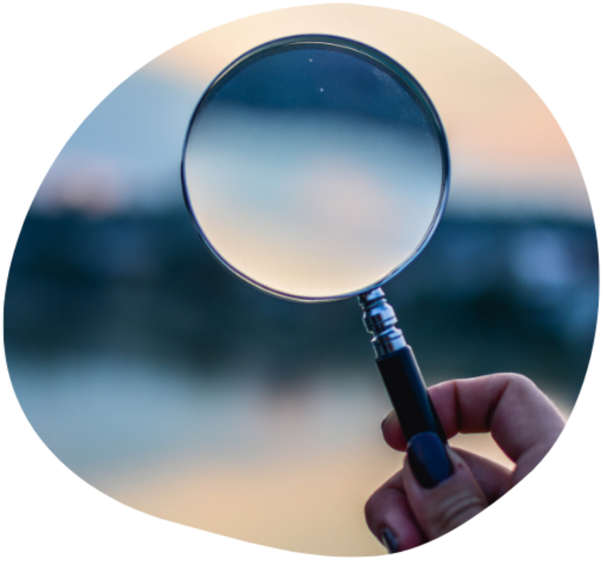

1) **In the reflection box below, write down what you noticed during or after anchoring yourself today.**
(This could be anything ranging from thoughts, feeling, behaviours, sounds around you, or changes within yourself)

2) **How effective were you in anchoring yourself in the present moment today?**
(0 = not effective at all, 100 = very effective)

Not effective at all at anchoring myself | Neutral | Very effective at anchoring myself

Change the slider above to set a response

reset

Submit

Save & Return Later

CM Module 3.

Flexible Thinking (Day 3)

Resize font:
⊞ | ⊟

This is your first **Thought Diary** activity for the program. In these Thought Diaries, you are encouraged to spend 5-10 minutes reflecting on the thoughts that you experienced today (particularly, those accompanied by strong or uncomfortable emotions!). Not only will you be noticing them, but you will be challenging them. We call this process "*catching*" your thoughts, where you pay attention to your thoughts and stop them in their tracks.

You might find that *thinking* about your *thinking* is a very unusual and difficult task - we are very rarely told to tune into our thoughts and challenge them in this way.

Keep in mind that the more you reflect on and challenge your thoughts, the stronger your flexible thinking will become, and the easier difficult emotions will be to manage. This is why we are asking you to complete this Thought Diary as many times as you can throughout this module.

You may like to complete each Thought Diary entry in the moment when you are having the thoughts (e.g., on your phone or note them down on a piece of paper and add them to the Thought Diary later). Or, you can complete your entry at the end of the day as a way of reflecting on the thoughts you had that day. Do what works best for you!

Note. If you felt that you only had positive thoughts today - that's great! You can still take part in the activity below.

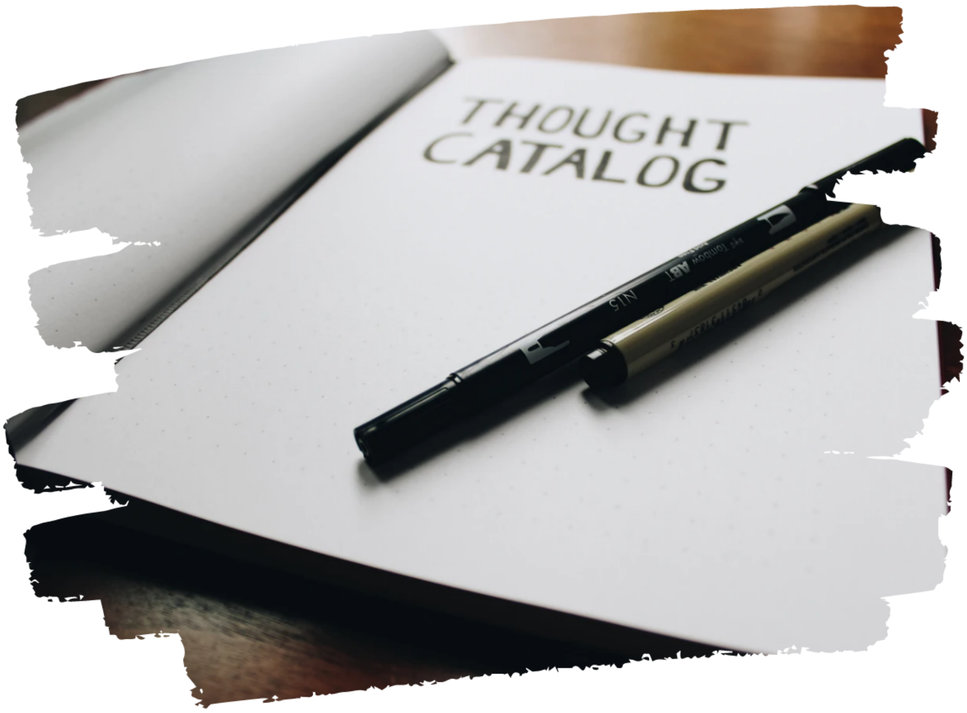

Click "**Watch video**" below if you would like to watch the summary video again.

Watch video

Click on the PDF link below to view the reading for this module again.

Attachment: Flexible Thinking Reading.pdf (2.44 MB)

What was a situation that you experienced today and what emotions did you experience?
(Reflect on a situation, positive or negative, that brought about certain thoughts. You can use the emotion wheel at the bottom of the page to help you to describe your emotions)

What automatic thoughts did you experience?
(These may be negative or positive! Sometimes we experience thoughts as images - you may like to describe an image)

Expand

Do these thoughts fall into a thinking trap?
(You can tick more than one box. Our thoughts often fall into more than 1 thinking trap! For a summary of the different thinking traps, click on the PDF link below)

- [ ] Jumping to conclusions
- [x] Catastrophising
- [ ] All-or-nothing thinking
- [ ] Tunnel vision
- [ ] "Should" statements
- [ ] I am not sure which thinking traps my negative thoughts fall into
- [ ] None of the above - I was experiencing positive thoughts!

Attachment: Thinking Traps List.pdf (0.05 MB)

Answer some of the following questions to help you to come up with an alternative, more balanced thought. If you have ticked multiple thinking traps, you do not need to answer all of these questions, pick 2 or 3 questions to answer which seem the most relevant and useful for you!

Am I 100% sure that these negative outcomes will occur? If I am not 100% sure, what are other possible outcomes?

Expand

If ___ does happen / is true, can I cope with it? What can I do to help myself cope with this?
(You may like to reflect on how you have coped/managed in the past in similar situations)

Expand

If ___ happens/is true, is it really as terrible as it seems? Are the consequences really so important?

Now that you have answered some questions above to challenge your thinking style, write down a more <u>balanced</u> and <u>realistic</u> perspective below:
(i.e, Another way to view/interpret the situation)

Expand

If you would like to a PDF summary of different questions to use to challenge your automatic thoughts, click on the link below.

Attachment: Questions for reframing thoughts.pdf (0.05 MB)

Part 2 - Alternative Actions (Day 12)

Resize font:

Page 1 of 1

In today's activity, spend 5-10 minutes to reflect on:
1) an EDB that you engaged with today (this might be the same as previous days or a new one)
2) whether you were able to replace it with a healthier alternative action.

Keep in mind that alternative actions *don't have to be big*; your alternative actions may involve making small behavioural changes.

Click on the PDF link below for a list of examples of possible alternative actions. Also, refer back to your saved responses for Day 8, where you made your own personal list of alternative actions. *(If you have not completed Day 8, we suggest that you go back and complete the activity for Day 8 first before completing today's activity).*

Attachment: Possible Alternative Actions.pdf (0.05 MB)

Click on the PDF link below if you would like to look over the reading again for Part 2:

Attachment: Part 2 - Changing EDBs Reading.pdf (0.82 MB)

Click "**Watch video**" below if you would like to watch the summary video for Part 2 again.

Watch video

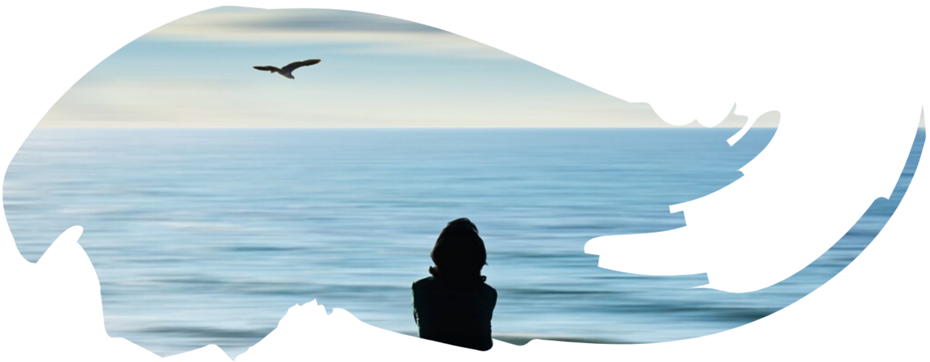

What was the EDB that you experienced today? Describe it below.
(E.g., ruminating, suppressing thoughts, avoiding people/places, yelling at someone etc.)

Expand

What was the situation that triggered the EDB today?

What emotions do you think were driving the EDB today?
(You can use the emotion-wheel at the bottom of the page to help you identify and describe the way you were feeling)

Expand

What alternative action did you use?
If you didn't try to replace your EDB with an alternative action, what could you have done instead? This is helpful to consider for the next time that you engage with this EDB.

Expand

What were the outcomes of the alternative action?
(E.g., how did it make you feel? How did it make others respond to you? Did you feel better or worse after using the alternative action?). If you didn't use an alternative action, write down what you expect the outcomes would have been.

Expand

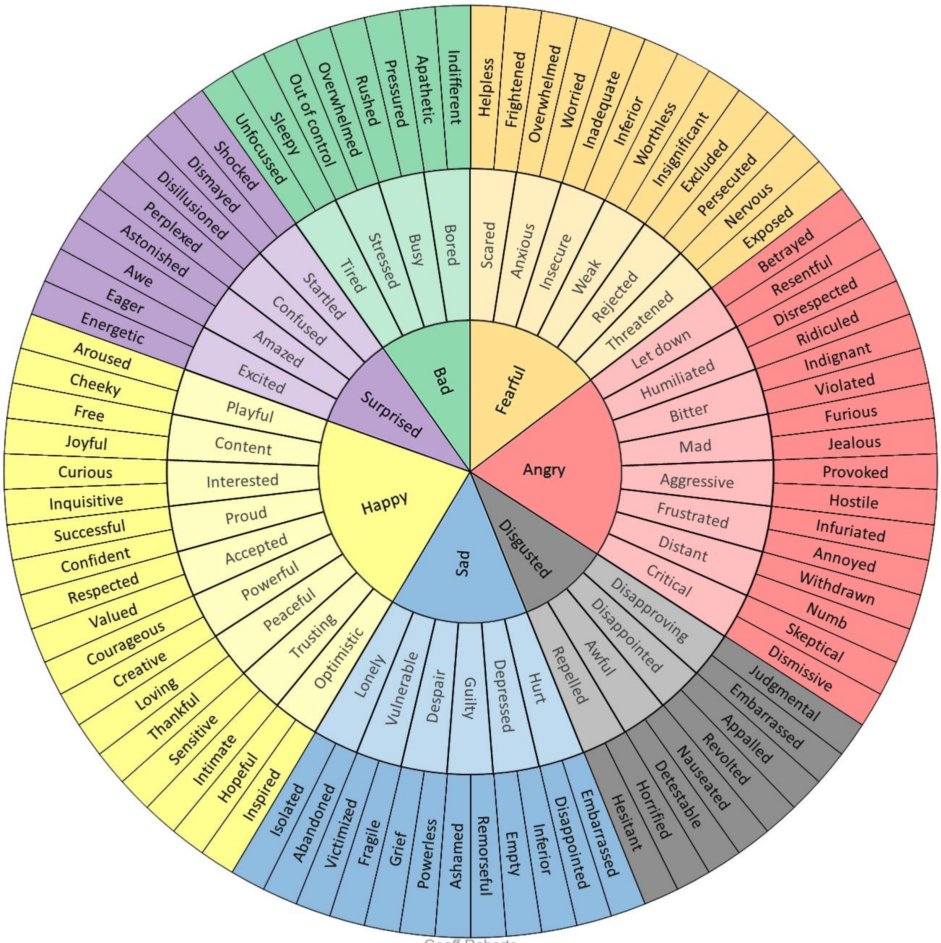

Geoff Roberts

CanCope Lifestyle – visual snapshot of example activities

CL Module 1.

Resize font:

Module 1: Eating Well After Treatment

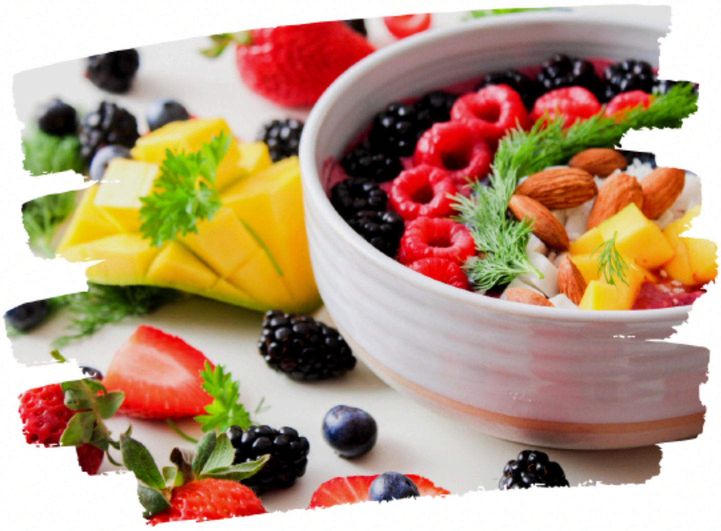

You were sent the Module 1: Eating well after treatment material 6 days ago. In this activity, reflect on your progress in engaging with healthy eating habits.

1) **Indicate below how you would describe your diet over the previous week:**

Very poor - I don't think I've eaten very healthily | Neutral | Very good - I think I have eaten very healthily!

Change the slider above to set a response

reset

2) **Describe below whether you applied the Module 1 Email material to your life. If so, describe what you did:**
(e.g., Did you engage in healthy eating habits throughout the week? Did you cook any of the recipes in the readings? Did you increase your fruit and vegetable intake? Etc...)

Expand

3) **Describe how you plan to engage in healthy eating habits over the next week:**
(E.g., Do you plan to make specific healthy recipes? Do you plan to increase your fruit and vegetable intake? Etc...)

Expand

Submit

Save & Return Later

CL Module 2.

Module 2: Staying Active After Treatment

Resize font:

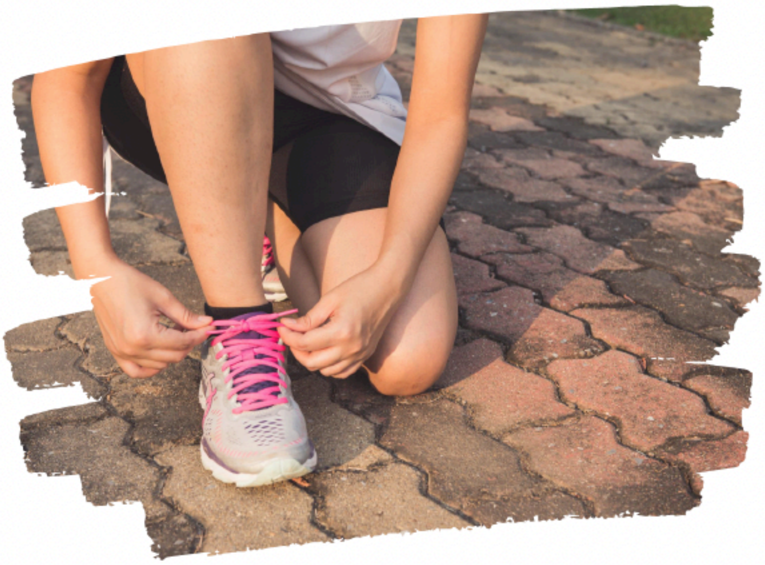

You were sent the Module 2: Staying active after treatment material 6 days ago. In this activity, reflect on your progress in staying active and engaging with physical activity.

1) **How content are you with your level of physical activity this week?**

Not very happy - I am not very happy with my physical activity level	Neutral	Very happy - I am very happy with my physical activity levels

Change the slider above to set a response

reset

2) **Describe below whether you applied the Module 2 Email material to your life. If so, describe what you did:**
(e.g., Did you engage with any of the physical exercises from the Email material? For example, did you engage with aerobic, strength, flexibility, or pevic floor exercises? How many times, for how long? Etc...)

Expand

3) **Describe how you plan to stay active over the next week:**
(E.g., Maybe you plan to schedule in some physical activity every day / every second day. Maybe there are certain new exercises you plan to try? Etc...)

Expand

CL Module 3.

Module 3: Relaxation for Mental Health

Resize font:
⊞ | ⊟

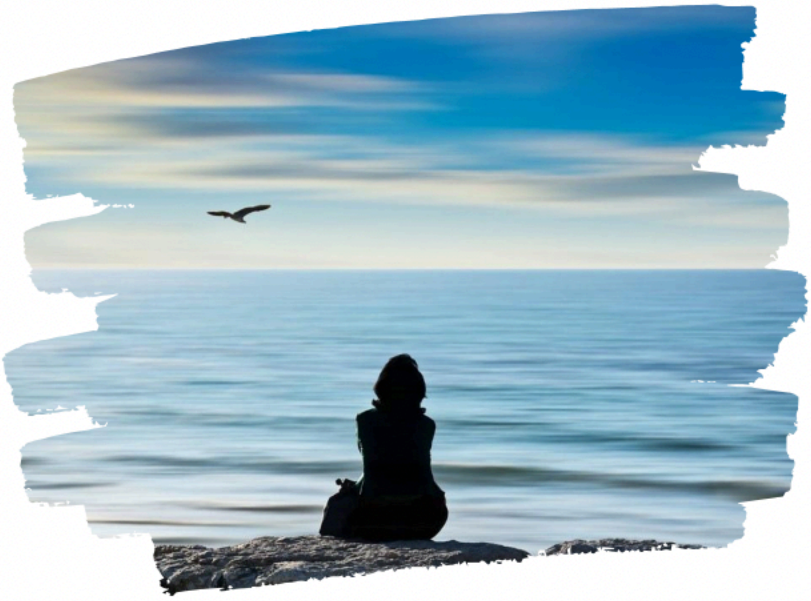

You were sent the **Module 3: Relaxation for mental health** material 6 days ago. In this activity, reflect on your levels of stress and whether you were able to spend time engaging with relaxing activities.

1) **How relaxed did you feel this week?**

Not relaxed at all | Neutral | Very relaxed

Change the slider above to set a response

reset

2) **Describe below whether you applied the Module 3 Email material to your life. If so, describe what you did:**
(e.g., Did you manage to engage with activities to help you relax throughout the week? What were those activities? Did you listen to any of the relaxation audios? How helpful were they? Etc...)

Expand

3) **Describe how you plan to engage with relaxing activities over the next week:**
(E.g., Maybe you plan to schedule in some daily "down time". Maybe you plan to engage with a new relaxation strategy, such as going for a slow walk, listening to music, doing Yoga. Maybe you plan to listen to a different relaxation audio? Etc...)

Expand

CL Module 4.

Module 4: Sleeping Well After Treatment

Resize font:

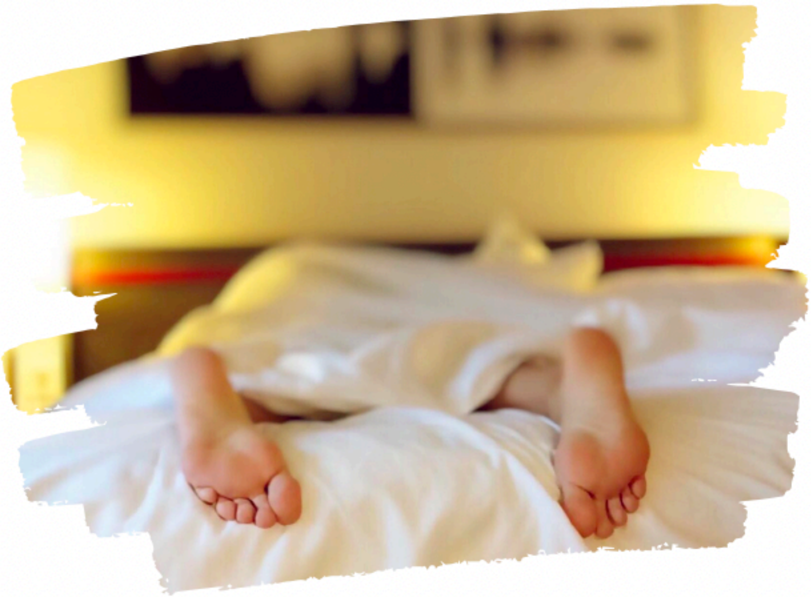

You were sent the **Module 4: Sleeping well after treatment** material 6 days ago. In this activity, reflect on your sleep and levels of fatigue. Also reflect on whether you were able to use the healthy sleep strategies outlined in the reading.

1) How would you describe your sleep over the previous week?

Very poor! I experienced many issues sleeping	Neutral	Very good! I experienced hardly any issues sleeping.

Change the slider above to set a response

reset

2) How would you describe your levels of fatigue over the previous week? (i.e., tiredness)

Very low - I haven't been fatigued/tired at all	Neutral	Very high - I have been very fatigued/tired

Change the slider above to set a response

reset

3) Describe below whether you applied the Module 4 Email material to your life. If so, describe what you did:

(e.g., Did you take into consideration some of the healthy sleep "hygiene" strategies - such as reducing caffeine, increasing light in the morning and decreasing light at night? Etc...)

Expand
